# Training Movement Velocity Significantly Affects the Performance of Myoelectric Control

**DOI:** 10.1109/TNSRE.2025.3610352

**Published:** 2025

**Authors:** Troy N. Tully, Amelia E. Nelson, Jacob A. George

**Affiliations:** Department of Biomedical Engineering, The University of Utah, Salt Lake City, UT 84132 USA; Department of Biomedical Engineering, The University of Utah, Salt Lake City, UT 84132 USA; Department of Electrical and Computer Engineering, The University of Utah, Salt Lake City, UT 84132 USA; Department of Physical Medicine and Rehabilitation, The University of Utah, Salt Lake City, UT 84132 USA; Department of Biomedical Engineering, The University of Utah, Salt Lake City, UT 84132 USA; Department of Mechanical Engineering, The University of Utah, Salt Lake City, UT 84132 USA

**Keywords:** EMG, myoelectric control, prosthetic control, kinematic regression, training data, training paradigm

## Abstract

Our native hands are uniquely capable of operating across a wide range of speeds and forces. In contrast, most commercial myoelectric prostheses typically provide limited speed and force output. One approach to endow myoelectric prostheses with variable speed and/or force output is to use continuous kinematic positions of the prosthesis based on electromyography (EMG). Within the field of machine learning, it is well established that homogeneous training data can lead to bias that negatively impacts the run-time performance of the algorithm. Yet, most continuous decoders are trained on a homogeneous dataset involving only a single kinematic speed. To this end, we systematically investigated how different training speeds influence myoelectric control with two common continuous decoders on multiple performance metrics. We compared a Kalman filter (KF) and Convolutional Long Short-Term Memory (C-LSTM) neural network trained on slow, medium, fast, and mixed-speed datasets, evaluating their performance in offline analyses and in two real-time online tasks with the user actively in the loop. We found that training speed significantly affected algorithm performance, but effects were often algorithm dependent. Linear algorithms, like the KF, are likely to exhibit lower unintended movement errors and smoother control when trained on slow-speed data but will also struggle to generalize to higher movement speeds. In contrast, nonlinear algorithms like the C-LSTM can likely provide greater adaptability, with mixed-speed training leading to improved accuracy and task success rates across conditions. Although an often-overlooked implicit parameter, these findings explicitly demonstrate that a lack of diverse training speeds in existing myoelectric control training paradigms leads to worse decoder performance. By incorporating a range of movement speeds into training protocols or decoder design, myoelectric continuous decoders could achieve more dexterous and robust control, potentially improving prosthetic usability and retention.

## I. Introduction

The current standard of care for upper-limb amputees remains unsatisfactory, with up to 50% of individuals abandoning their prostheses, citing poor and unreliable control as a primary reason [1], [2], [3]. Most myoelectric prostheses use pattern recognition to decode multiple movements from electromyography (EMG) activity, enabling discrete gesture classification for real-time applications [4], [5], [6], [7], [8]. Despite near-perfect performance, these systems are inherently limited by their reliance on single discrete class predictions, which restricts proportional control necessary for fine force modulation. Users must transition through sequential actions, which can limit the fluidity of movement and any ambiguity during class transitions reduces speed and stability when signals fall between classes [9]. In contrast, simultaneous and proportional control of multiple degrees of freedom has been shown to outperform classification-based control in upper-limb prosthetics during real-time tasks [10]. Recent efforts in pattern recognition are beginning to address this limitation by specifically using error during model transitional states between gestures as a primary indicator of performance [11].

In contrast, our native hands are uniquely capable of operating across a wide range of speeds and forces [12], [13]. One approach to provide myoelectric prostheses with variable speed and/or force output is to utilize a continuous decoder, supervised machine learning algorithm, in which EMG signals are correlated with the user’s motor intent to provide continuous proportional control [4], [14]. Indeed, amputees have shown a significant preference for the quickness and control of speed and force afforded by proportional control [15].

Continuous decoders for myoelectric control rely on a labeled dataset where continuous EMG signals are mapped to continuous kinematic positions, velocities, or forces [14], [16], [17], [18], [19], [20], [21]. The quality and diversity of the training data are critical to the run-time performance of any supervised machine learning algorithm, and this holds true for continuous decoders for myoelectric control as well [22], [23]. Strikingly, most continuous decoders for myoelectric control are trained on fixed-speed datasets and thus disregard this important and fundamental tenet of supervised learning [4], [14], [16], [19], [23], [24], [25], [26], [27]. Fast, forceful movements are characterized by large and distinct EMG patterns, whereas slow movements generate more subtle and noisier differences in EMG [28], [29], [30]. To the extent that the dataset incorporates different training speeds, the more diverse the underlying EMG will be, which in turn may impact the overall performance and ability of a continuous decoder to generalize across the range of forces and speeds encountered in daily activities [31].

Here, we explore the impact of training speed on two commonly used continuous decoders: a linear Kalman Filter (KF) [14], [23], [26], [32] and a nonlinear Convolutional Long Short-Term Memory (C-LSTM) neural network. We first show how training at different fixed movement speeds influences algorithm accuracy across a range of testing speeds in an offline analysis. We then demonstrate how fixed training speeds impact the real-time performance of closed-loop myoelectric tasks with a human actively in the loop. We evaluate this online control for a target-tracking task that demands precise proportional position control, as well as a novel catching task designed to emphasize rapid proportional position control. Together, the findings demonstrate the need to incorporate diverse training speeds in existing training paradigms to ensure continuous decoders for myoelectric control can replicate the diverse speeds and forces of the natural human hand.

## II. Methods

### A. Human Subjects

Twenty-five healthy participants were recruited for this study. All participants were between the ages of 18 and 32 years old. Eight participants for the offline analysis, twelve for the Target-Tracking Task (six per algorithm), and five participants for the Block Catching Task. Informed consent and experimental protocols were carried out in accordance with the University of Utah Institutional Review Board 00098851 and the Declaration of Helsinki.

### B. Data Acquisition

Surface electromyographic (sEMG) signals were recorded using the Summit Interface Processor (Ripple Neuro LLC, Salt Lake City, UT, USA). A custom-made neoprene sleeve embedded with 34 brass-coated marine snaps were positioned on the participant’s forearm, such that 32 dry electrodes were placed over the extrinsic hand muscles, with one electrode serving as an electrical reference and another as ground. Continuous EMG signals were sampled at 1 kHz across all 32 channels.

### C. EMG Preprocessing

Raw EMG signals were band-pass filtered using a sixth-order high-pass Butterworth filter at 15 Hz and a second-order low-pass Butterworth filter at 375 Hz to isolate the frequency range of interest. Notch filters were applied at 60, 120, and 180 Hz. Differential EMG signals were calculated for all possible pairs of channels, resulting in 496 (32 choose 2) differential recordings. The mean absolute value (MAV) was computed from both the 32 single-ended channels and the 496 differential recordings using an overlapping 300-ms window updated at 30 Hz. The resulting EMG feature set consisted of 528 smoothed MAV features, which were used as input to the decoding algorithm [14], [33].

### D. Experimental Design

To evaluate the effects of training speed on myoelectric control, we conducted one offline analysis and two online analyses using continuous decoders. Specifically, we assessed the performance of a Kalman Filter (KF) and a Convolutional Long Short-Term Memory (C-LSTM) neural network under different training conditions. Offline analysis focused on model accuracy in predicting kinematic position, while online analyses assessed real-time control performance in two virtual tasks: the Target-Tracking Task (TTT) and the Block Catching Task (BCT).

### E. Training Data

For the offline analysis, participants performed myoelectric training for a three-degree-of-freedom (DOF) virtual hand and wrist while wearing the sEMG sleeve. Participants were instructed to mimic the preprogrammed movements of the virtual hand displayed on a computer monitor with their right hand. The three DOFs of the virtual hand were: 1) grasping and opening the hand (i.e., simultaneous flexion/extension of digits 1 through 5), 2) wrist flexion/extension, and 3) wrist pronation/supination. These three DOFs match the most common DOFs in existing prostheses and align with patient priorities [34]. Notably, each DOF is bidirectional, giving rise to a total of six different movements. Kinematics of each DOF were normalized from −1 to + 1 such that −1 represents maximum opening/extension/supination, + 1 represents maximum grasping/flexion/pronation, and 0 represents a neutral baseline resting position. Training datasets were collected as participants mimicked either slow, medium, or fast movements.

Slow movements consisted of a 3-second movement to the kinematic endpoint, followed by a 1-second hold at the kinematic endpoint, and then a 3-second return to the kinematic resting point, for a total movement duration of 7 seconds. Medium movements consisted of a 1.5-second movement to the kinematic endpoint, followed by a 1-second hold at the kinematic endpoint, and then a 1.5-second return to the kinematic resting point, for a total movement duration of 4 seconds. Fast movements consisted of a 0.5-second movement to the kinematic endpoint, followed by a 1-second hold at the kinematic endpoint, and then a 0.5-second return to the kinematic resting point, for a total movement duration of 2 seconds. Because slow movements naturally yield more training data than fast movements, we included a sufficient number of trials (12 in total) to ensure the dataset contained a surplus of data, making any bias due to data quantity negligible. Each training dataset included 20 trials across the six movements, resulting in a total of 120 trials per dataset. Given the extensive amount of data collected, breaks were given between the collection of each dataset to minimize participant fatigue.

For the online TTT, participants performed the same six movements as the offline analysis. Participants conducted two training sessions for each speed. Each training contained six trials across each movement, resulting in 36 trials per session for a total of 72 training trials per speed.

For the BCT, participants performed the task with control over one DOF of the hand (grasping and opening). Training sessions included six trials across each movement, resulting in 12 trials per session, performed twice for a total of 24 training trials per speed. For all training sessions described above, each training speed condition was done in a pseudorandomized, counterbalanced order to eliminate the possibility of any order effects.

### F. Continuous Decoders

Two different continuous decoders were used in this study to see how different training speeds impacted simple linear decoders and more complex, nonlinear decoders. The first decoder was a KF. The formulation of the KF for control was originally described in Wu et al. [35], its adaptation for EMG control is described in Nieveen et al. [26], and the ad-hoc modification has been described in George et al. [14]. To keep it brief here, a summary of the formulation can be found in the supplementary methods. In this work, we implement the KF as a continuous decoder. The KF uses a recursive Bayesian framework to continuously update its estimate of the user’s intended position by combining current EMG observations with prior predictions of state dynamics. When the observation model (i.e., the likelihood of EMG activity given kinematic position) and the state model (i.e., how kinematics evolve over time) are assumed to be linear and Gaussian, the KF provides an analytically optimal estimate of motor intent. This recursive formulation enables real-time control and naturally smooths noisy EMG inputs, resulting in stable and proportional predictions of kinematic position [36]. For online analyses, a Modified Kalman Filter (MKF) was used [14]. The modification affects state behavior through gain and values that edit the state between iterations during online use. No modifications to the gain were used in this study, but a 20% threshold was used, such that any kinematic predictions below 20% of the full kinematic range were set to zero, and anything above 20% was rescaled between 0 and 100% of the full kinematic range.

The second algorithm used in this study was a C-LSTM. This network follows a standard C-LSTM hybrid widely used in EMG decoding and is built upon previous work [37]. It leverages shallow temporal/feature convolutions to extract local motifs that are then integrated by an LSTM to model temporal dependencies, before fully-connected regression produces proportional kinematics. This mirrors prior continuous-decoding LSTM pipelines [38], while remaining architecturally analogous to position-aware RCNN prosthesis controllers [39], which likewise combine convolutional front-ends with recurrent memory. Reviews of EMG-HMI methods place such CNN-RNN hybrids as a canonical design pattern, supporting the view that these architectures are fundamentally similar in structure and function [40], [41]. This model was designed to predict kinematic positions by capturing both spatial and temporal dependencies within sequential EMG data. The input data, organized into sliding windows of 10 samples, was first processed by convolutional layers that extracted spatial features using 10 filters of size [32, 1]. These convolutional layers included batch normalization and ReLU activation to enhance feature extraction and stability. The convolutional output was then passed through additional convolutional layers with stride-based dimensionality reduction to refine the feature maps.

Following spatial feature extraction, the data was fed into one LSTM layer with 40 hidden units, which modeled temporal dependencies by leveraging the sequential nature of the EMG signals. The architecture also included fully connected layers to map learned representations to kinematic predictions. The model was trained using Stochastic Gradient Descent with an initial learning rate of 0.0002 for up to 1000 epochs, with a patience of five epochs for early stopping based on validation performance to prevent overfitting. This hybrid architecture effectively combined the convolutional layers’ spatial learning capability with the C-LSTM layers’ temporal modeling to enhance kinematic prediction accuracy.

### G. Offline Analysis

Each of the two training decoders were trained on a total of seven different training datasets: 1) slow movements, 2) medium movements, 3) fast movements, 4) slow + medium movements (S+M), 5) slow + fast movements (S+F), 6) medium + fast movements (M+F), and 7) slow + medium + fast (All) movements. For each training condition and algorithm, 6-fold validation was used for offline analysis of machine learning performance. An equal number of trials were used from each of the training sessions to minimize any order effects that may have appeared in the data. The trials were shuffled so the testing and training data varied from each training session per validation fold. For analysis, decoders were trained using 12 of the 20 trials from a single speed or a mix of two or three speeds. The remaining eight trials were used for testing. For the C-LSTM, two trials were used for validation to reduce overfitting. Each of the seven training datasets was individually processed by each algorithm, formulating 14 unique myoelectric control decoders. These decoders were subsequently evaluated on the remaining testing datasets for the slow, medium, and fast conditions, for a total of 42 outcomes per participant (14 training conditions x 3 testing conditions). Outcomes measured myoelectric algorithm performance based on the root mean squared error (RMSE) between algorithm predictions and ground-truth target kinematics that the participants attempted to mimic. RMSE was calculated from the normalized range of motion and divided into two metrics: intended movement RMSE and unintended movement RMSE (i.e., cross-talk) [14]. Intended movement RMSE captures the ability of an individual to precisely control a given degree of freedom. Unintended movement RMSE captures the ability of an individual to move a given degree of freedom in isolation. The mean performance across the three testing conditions (slow, medium, and fast) was determined for each algorithm and each metric for statistical analyses.

### H. Target-Tracking Task

Adapted from previous work [14], [26], [37], [42], we used a virtual TTT to quantify user and algorithm performance. This task involves controlling a virtual bionic arm (MSMS; John Hopkins Applied Physics Lab, Baltimore, MD, USA), where the participant is provided real-time visual feedback. In this task, the participant actively controlled the virtual MSMS hand and attempted to move select DOFs to a target location while holding the remaining DOFs at rest. Target locations for the intended DOFs were continually updated such that the target velocity was constant from rest to the maximum amount of flexion/extension possible and back to rest to evaluate proportional control for the selected DOF, while the remaining DOFs had targets at rest. The participant was instructed to hold all DOFs within their target location for the trial duration and viewed the target locations and hand kinematics in real-time on a computer screen. One target was shown for each DOF, and if the corresponding kinematic was within ±15% of the target location, the target would change color. A red target indicated they were further than ±15% from the target, while a green target indicated they were within ±15% of the target.

Each of the two training decoders were trained on a total of five different training datasets: 1) slow movements, 2) medium movements, 3) fast movements, 4) slow + fast movements, and 5) slow + medium + fast movements. Each participant controlled the virtual hand with each algorithm to perform the target movements at three velocities, totaling 15 sessions. Each session included four trials per movement across six movements, amounting to 24 trials. Decoders were blinded from the participants and appeared in a pseudorandomized, counterbalanced order to minimize learning effects within a session. Testing speeds were also pseudorandomized and counterbalanced to minimize the possibility of any order effects.

For the TTT analysis, we compared the algorithm performance of the 10 decoders on the TTT using two metrics: 1) intended movement RMSE [42], and 2) the log mean absolute jerk [26], [43], [44]. Intended RMSE was calculated as done for the offline analysis, except the RMSE was calculated from the 15%-error window, such that the RMSE was zero if the DOF was anywhere within the 15%-error window (consistent with the visual feedback the participant received). The mean intended RMSE across the three testing conditions was determined for each algorithm for statistical analyses. Due to the inherent limitations of using RMSE [45], we analyzed Log Mean Absolute Jerk (LMAJ) on the TTT to assess the precision and smoothness of the decoder. LMAJ was calculated using the predicted kinematics for the intended DOF during each trial on each testing dataset. The median LMAJ across the three testing conditions (slow, medium, and fast) was determined for each algorithm for statistical analyses. Additionally, Statistical non-Parametric Mapping (SnPM) [46], [47], [48], [49] two-tailed t-test was performed between target and predicted kinematics to temporally assess the decoder’s performance across different phases of each movement.

### I. Block Catching Task

Most myoelectric control tests emphasize precision and dexterity rather than ballistic-speed movements. To probe rapid proportional position control, we developed a virtual Block Catching Task (BCT) for this study. Unlike the TTT’s steady-velocity precision demands, the BCT imposes ~100 ms reaction windows and large-amplitude commands to a moving target, directly testing algorithm responsiveness and user coordination at ballistic speeds. Unlike the TTT, this task involved controlling only one DOF of a 3D-rendered virtual bionic hand (MuJoCo, Google DeepMind, London, UK) to open and close its grip and catch a falling virtual block. Difficulty levels (easy, medium, hard) were set by varying block length, providing approximately 400, 200, and 100 milliseconds to catch it, respectively. Each of the two training decoders were trained on a total of five different training datasets: 1) slow movements, 2) medium movements, 3) fast movements, 4) slow + fast movements, and 5) slow + medium + fast movements. Each participant controlled the virtual hand with each algorithm and attempted each of the three difficulties at a total of six times each. Decoders were blinded from the participant and appeared in a pseudorandomized, counterbalanced order to eliminate the possibility of any order effects. Each trial set included three practice attempts before three recorded attempts, where total successful catches were measured.

For the virtual BCT, the percentage of successful catches was used to quantify algorithm performance, providing an additional outcome measure beyond RMSE to evaluate overall control performance. Performance for all three difficulties was pooled for statistical analysis to assess overall performance across variable speeds.

### J. Statistical Analysis

Data were screened for normality using the Anderson-Darling test. A one-way analysis of variance (factor: training speed) was performed separately for each algorithm for each outcome metric. If a significant effect was observed, subsequent post-hoc unpaired t-tests were performed with Dunn-Sidak’s correction for multiple comparisons. As our data were normally distributed and the primary objective was an inferential comparison of group means across training speeds, results are reported as mean ± SEM to convey uncertainty in the mean estimate [50].

## III. Results

### A. Speed of Training Significantly Affects Offline Accuracy

Continuous decoders for myoelectric control allow for proportional position control that allows users to produce a range of output forces and speeds [15]. The quality and diversity of the training data is critical to the run-time performance [22], yet most continuous decoders are trained on a fixed-speed dataset that is not representative of the variable speeds users employ in daily activities [4], [14], [19], [23], [24], [25], [26], [27]. To this end, we tested the accuracy of continuous decoders for myoelectric control trained on a fixed speed when extrapolated to novel, unseen speeds.

**Fig. 1.**
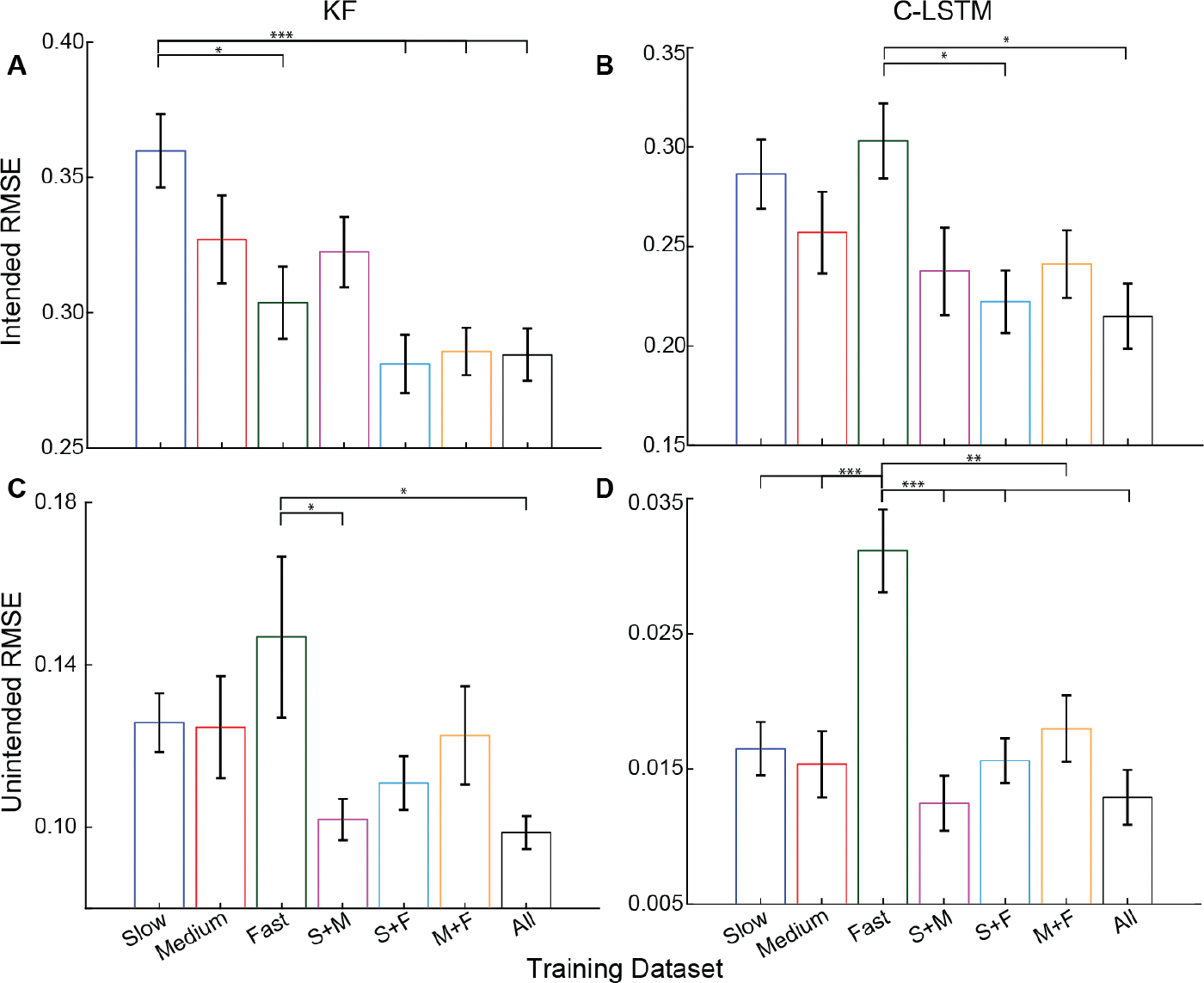
Impact of training dataset speed on algorithm performance. Performance is assessed via intended movement RMSE (top) and unintended movement RMSE (bottom). Slow training speeds resulted in worse intended movement RMSE for the Kalman filter (A), whereas fast training speeds resulted in worse intended movement RMSE for the C-LSTM (B). Fast training speeds also resulted in significantly worse unintended movement RMSE, and this was true for both the KF (C) and the C-LSTM (D). Single, double, and triple asterisks denote statistical significance at corrected alpha levels of 0.05, 0.01, and 0.001, respectively. Data show the mean ± SEM for the eight participants (N = 8). All data screened for normality using Anderson-Darling test, all *p* > 0.05.

For both a linear Kalman Filter (KF) and a nonlinear Convolutional Long Short-Term Memory (C-LSTM) neural network, the speed at which the training data was collected had a significant effect on the algorithm accuracy, as assessed by the RMSE of intended movements (*p*’s <0.01, separate one-way ANOVAs). For the KF, post-hoc pairwise comparisons revealed that slow training speed resulted in significantly worse performance relative to all the other conditions except the medium condition and the combined slow + medium (S+M) condition (Fig. 1A). In contrast, for the C-LSTM, the fast training speed resulted in worse performance compared to the slow + fast (S+F) condition and the condition with all the speeds (Fig. 1B). Thus, the impact of training speed is dependent on the algorithm, but nonetheless substantial. For both decoders, the best performance was achieved when more diverse data was present (i.e., the condition with all the speeds or the condition with slow + fast speeds).

### B. Fast Training Data Increases Unintended Movements in Offline Analyses

Homogeneous training data that is not representative of the testing conditions can lead to algorithm bias that negatively impacts performance. For myoelectric prostheses, algorithm bias can lead to unintended movements of the prosthesis, which have been shown to cause prosthesis abandonment [2]. To this end, we examined how training on different fixed-speed datasets impacts the unintended movements produced by the decoders.

For both the KF and C-LSTM, training speed had a significant impact on unintended movements (*p*’s ‘! 0.05, separate one-way ANOVAs). Post-hoc pairwise comparisons showed that fast training speed resulted in significantly more unintended movements (Fig. 1C and 1D). For the C-LSTM, the fast training speed was significantly worse than all other conditions (Fig. 1D), but for the KF the fast training speed was significantly worse than the slow + medium (S+M) condition and the condition with all speeds. These results suggest that, regardless of the algorithm, slower training speeds may help reduce the unintended movements of continuous decoders for myoelectric control decoders.

### C. Diverse Training Speed Improves Online Control Accuracy

The offline analyses above demonstrate that training speed can have a significant impact on algorithm performance, however, prior work has also shown that offline performance cannot directly predict online performance when a user is actively in the loop [5], [45], [51], [52]. Thus, as a next step, we recruited 12 participants to complete an online Target-Tracking Task (TTT) with real-time visual feedback. Training speed did not have a significant impact on the RMSE on the modified KF (MKF), although there was a trend (*p* =0.15, ANOVA). For the C-LSTM, training speed had a significant impact on RMSE (*p* <0.01, ANOVA), with slow-speed training performing significantly worse than the all-speed training (*p* <0.05, subsequent pairwise comparisons). Additionally, SnPM analyses showed most error occurred during the transition phases during the TTT and the fast testing had the highest error during transition phases, suggesting faster movements are more difficult to reproduce (Fig. S2 & S3). Most notably, the C-LSTM shows improved performance across transition phases when trained on all speeds (Fig. S3). Together, these findings further support the idea that diverse training speeds can improve algorithm performance.

### D. Slower Training Speeds Lead to Smoother Control for the MKF, but Not the C-LSTM

**Fig. 2.**
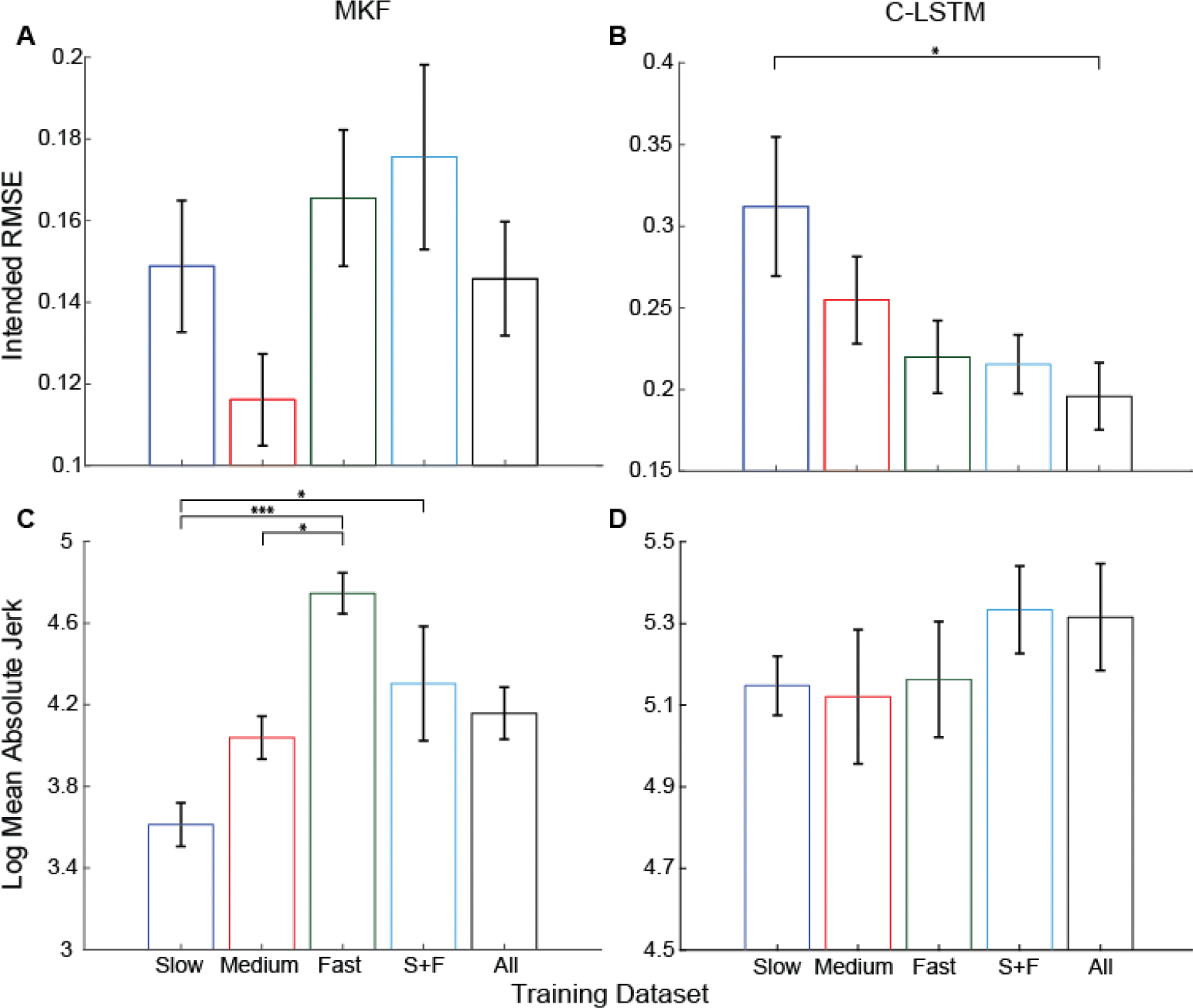
Impact of training dataset speed on target-tracking task performance. Performance is assessed via intended movements RMSE (A+B) and LMAJ (C+D). In general more diverse training speeds led to better performance. Training speed had no significant effect on the RMSE of the MKF (A). However, slow training data for the C-LSTM led to significantly worse RMSE than the combined training speeds condition (B). Fast training speeds led to significantly worse LMAJ for the MKF (C), where training speed had no impact on the C-LSTM (D). Single, double, and triple asterisks denote statistical significance at corrected alpha levels of 0.05,0.01, and 0.001, respectively. Data show the mean ± SEM for the six participants (N = 6). All data screened for normality using Anderson-Darling test, all *p* > 0.05.

Myoelectric decoders must be accurate and precise to generate fluid and consistent movements you would expect when performing delicate tasks. Having assessed accuracy with RMSE and due to the limitations of using only RMSE [45], we next sought to investigate each algorithm’s precision. To quantify precision, we analyzed the Log Mean Absolute Jerk (LMAJ) of their kinematics during the TTT. Training speed significantly impacted LMAJ with the MKF (*p* <0.001, ANOVA). Post-hoc pairwise comparisons revealed that the slow training condition resulted in significantly smoother movements compared to the fast and the combined slow + fast (S+F) training (Fig. 2C). Additionally, the fast training speed was significantly less smooth than medium training speed (Fig. 2C). Visual observation of the kinematic traces (Fig. S1) confirmed the smoother trajectories found by this analysis. These results suggest that incorporating slower speeds into training can improve the smoothness of kinematic trajectories predicted by the MKF (Fig. S1). However, in contrast, the C-LSTM showed no significant differences in LMAJ across training speeds (Fig. 2D; *p* =0.65, ANOVA), suggesting that the smoothness of the C-LSTM is less dependent on the speed at which the training data was collected.

### E. Faster Training Speeds Improve Performance on Quick and Coordinated Tasks

**Fig. 3.**
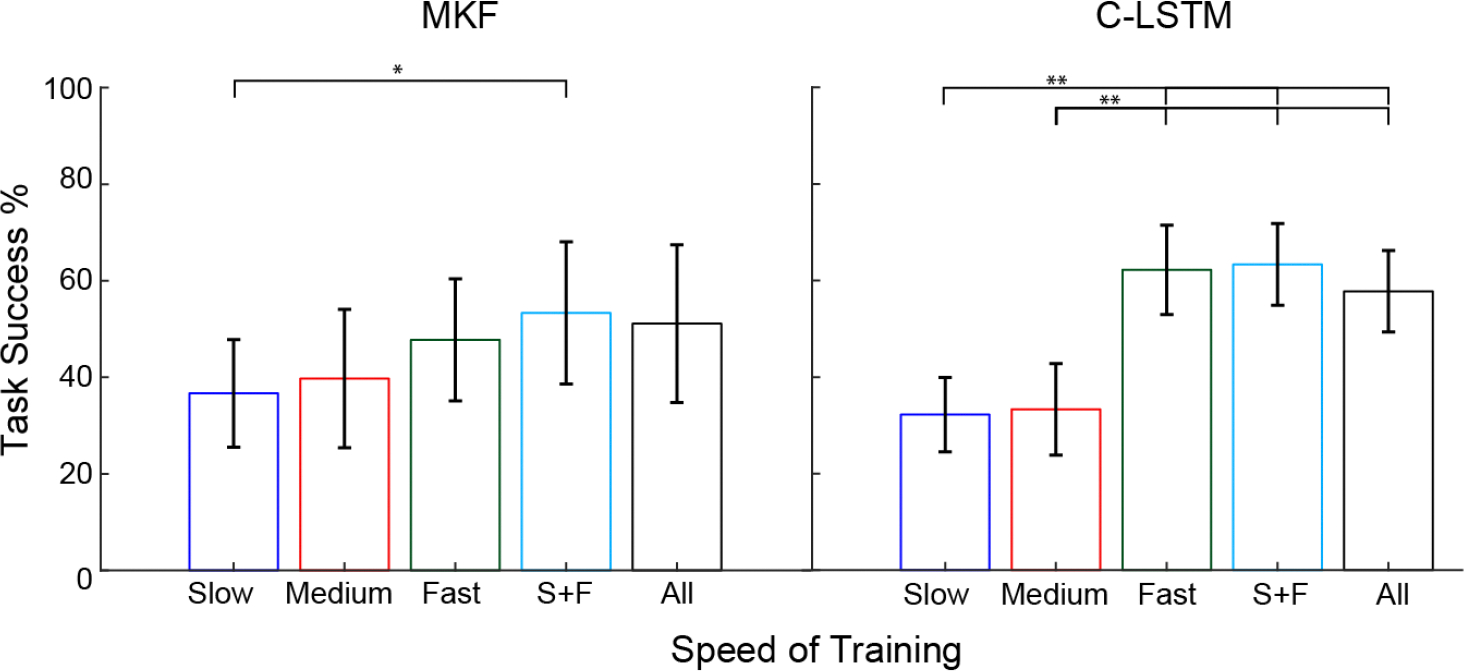
Impact of training dataset speed on block catching task. In general, faster training speeds led to better performance. For the MKF, slow + fast training data led to significantly higher success relative to the slow training. Similarly, with the C-LSTM, conditions including fast training speeds outperformed the slow and medium training conditions. Single, double, and triple asterisks denote statistical significance at corrected alpha levels of 0.05,0.01, and 0.001, respectively. Data show the mean ± SEM for each of the three attempts from the five participants (N = 15). All data screened for normality using Anderson-Darling test, all *p* > 0.05.

Accurate and smooth control is critical for delicate tasks, but other functional activities demand fast reactions. Tasks requiring responsiveness and high-speed coordination may benefit from a different blend of training speeds. To examine this, we evaluated the impact of training speed on real-time online performance in a virtual Block Catching Task (BCT). For both the MKF and C-LSTM, training speed significantly impacted task success rate (Fig. 3; *p*’s <0.05, ANOVA). For the MKF, the combined slow + fast (S+F) training condition led to significantly better performance compared to slow-only training. Similarly, for the C-LSTM, slow + medium (S+M) training speeds resulted in significantly worse performance compared to conditions incorporating fast training data, which included the fast, S+F, and all-speed conditions. As one might expect, incorporating task-specific training data improves task performance. In this case, the findings explicitly demonstrate that collecting training data at fast speeds can enhance performance in quick and coordinated tasks.

## IV. Discussion

In this study, we systematically investigated how training speed influences myoelectric control performance, focusing on two different continuous decoders, a Kalman Filter (KF) and a Convolutional Long Short-Term Memory (C-LSTM) neural network. We found that the velocity of training data significantly impacted algorithm performance across a range of offline and online metrics. As might be expected, incorporating task-specific training data led to better task performance, and when task conditions varied, incorporating diverse training data led to more accurate and robust performance. Notably, this is in direct contrast to the overwhelming majority of studies that utilize only a single fixed speed in their training data [4], [14], [19], [23], [24], [25], [26], [27]. As such, this paper serves as explicit documentation for researchers to incorporate more variable kinematic speeds in their training data to optimize the performance of continuous decoders for myoelectric prostheses.

One practical reason why prior works have not been explicitly trained on a variety of different speeds is that most training is performed under a mimicked training paradigm in which the ground truth kinematics are determined by the preprogrammed movement of a virtual or physical prosthesis. Programming in a variety of speeds is time consuming. Similarly, manually adjusting a fixed training speed to match what would be used in a functional task is also laborious, and, in many cases, the ideal speed for a given functional task is unclear. To this end, this work corroborates our prior work which recommended mirror training for unilateral amputees, where the ground truth kinematics are instead determined by motion capture of the contralateral limb during bilaterally mirrored movements [22]. Under this approach, the participant can explicitly collect training data at the speed they find most suitable for the task. Indeed, this element of mirror training may have been the predominant contributing factor to the improvements seen in this prior work [22].

The modified KF used in this paper is based on a linear Kalman filter that assumes the data are linear and Gaussian. As such, previous studies have shown that the KF performs best with structured, low-variance data [22]. Surprisingly, in this work, the KF demonstrated a notable ability to learn effectively from datasets containing multiple velocities. Although the KF still showed fewer unintended movements and smoother control (lower log mean absolute jerk) with slow-speed training, it achieved substantially better generalization and improved task success when trained on mixed-speed datasets compared to single-speed datasets. Prior work has hypothesized that improvements in KF performance with diverse training data may be the result of a lower Kalman gain [42]. More variability in the training data leads to less certainty among the electromyography (EMG) features; this is parameterized as a lower Kalman gain, which means the current state estimate has more weight relative to the new state predicted by EMG data [42]. Conversely, the C-LSTM consistently benefited from training with varied velocities, showing enhanced adaptability and robust performance across all metrics. The C-LSTM improvements are likely attributed to the ability of the nonlinear neural networks to capture intricacies of complex EMG signals. Although we validated this work with multiple decoders, the use of a novel C-LSTM may have impacted the result herein and future work should validate these findings with additional decoders.

An important question that can be raised is where does the error come from temporally during testing trials. Our SnPM analysis localized errors to movement transitions where the MKF showed clustered error on the return to rest at slow tests, DOF-specific localization at medium tests, and widespread phase-spanning error at fast tests. Similarly, the C-LSTM showed the smallest, offset localized error at slow tests, the most widespread error at fast tests, and the least temporal specificity when trained on multiple speeds.

These findings support recent efforts in pattern recognition where researchers have begun to prioritize class transition accuracy when comparing algorithm performance [11]. In contrast to continuous decoders, which are trained and evaluated on time-evolving EMG that includes transitions, pattern recognition systems are trained on static contractions and impose proportionality by scaling velocity with MVC [53]. This design creates a mismatch between the training data and the speed of execution and contraction level during online use. Prior pattern recognition studies demonstrate that MVC-based proportional control is affected by contraction strength and suggest collecting training data wherein the dataset contains a spectrum of voluntary contraction intensities to improve classification accuracy during online use [11], [54]. Taken together with our SnPM findings, our observations support the idea that transitions between classes are difficult for both proportional and pattern recognition systems, and these machine learning techniques require extensive training data to map transition periods accurately during online use [22]. Future studies should investigate pattern recognition control systems with functional tasks and simultaneous and proportional control of multiple degrees of freedom under ballistic movements to investigate the effect that training and testing speed might have on class transition error.

Future work should also corroborate these findings with upper-limb amputees performing activities of daily living. It is likely that the optimal training speed is unique to each patient and their preferred activities. Furthermore, we did not collect hand dominance and although hand dominance has been shown to not play a role in an offline analysis, future work should investigate the interplay of training speed and hand dominance [55]. Nonetheless, this work demonstrates that training speed is a critical factor for ensuring a properly calibrated myoelectric prosthesis. More broadly, as machine learning becomes increasingly used with myoelectric prostheses, this work serves as a critical reminder that the underlying training data should not be overlooked. In fact, this paper is yet another example where the training data is more important than the machine learning algorithm used [22], [23], [36], [42], [56], [57], [58], [59].

## Supplementary Material

supp1-3610352
