# Supplementary material for: Training Movement Velocity Significantly Affects the Performance of Myoelectric Control
Source: IEEE Trans Neural Syst Rehabil Eng. Author manuscript; Available in PMC 2025 Oct 30. (PMC12574017; doi:10.1109/TNSRE.2025.3610352)
Supplement: supp1-3610352 [file NIHMS2114114-supplement-supp1-3610352.docx]

1. **Supplementary Figures**


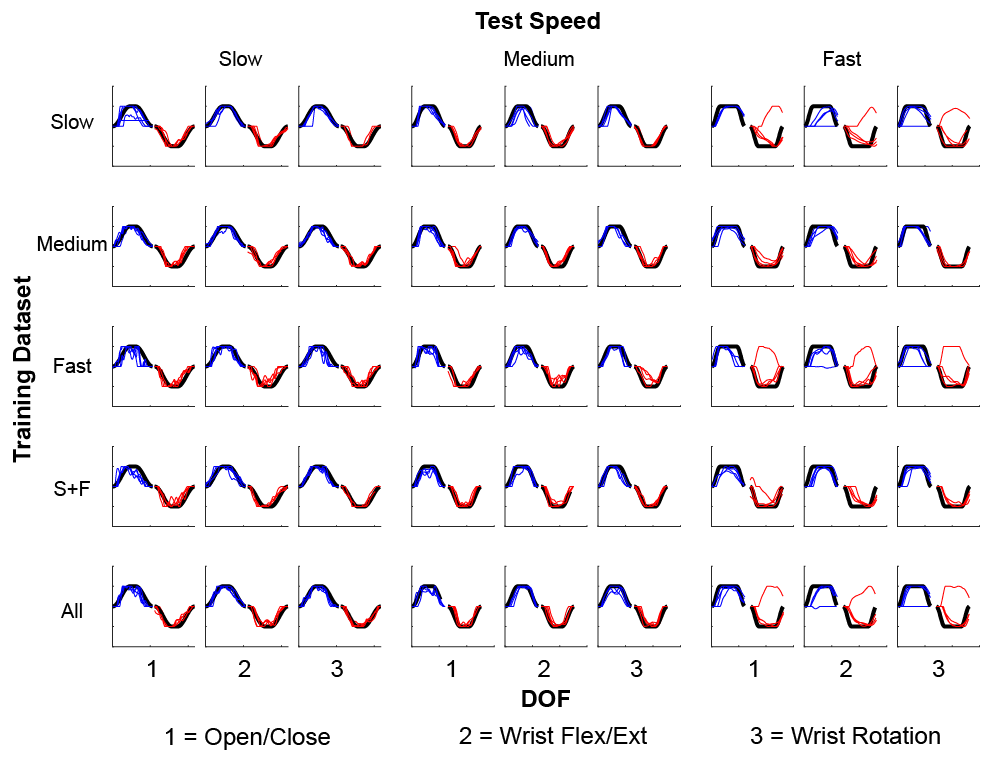


**Supplementary Fig. 1.** Time-normalized kinematic traces show MKF-predicted kinematics across all conditions for training and testing for each bidirectional degree of freedom (DOF) from one participant during the TTT. Traces from fast training were notably unsmooth during the slow test compared to other training conditions, while slow training generated smoother trajectories but introduced substantial lag when tested at fast speeds. Medium training speeds yielded comparatively stable trajectories and improved performance on fast tests compared to the slow training dataset. Traces from combined datasets show mixed performance across metrics and test conditions. Black trace is the target kinematic, while blue and red traces show predicted kinematics during flexion and extension trials, respectively.


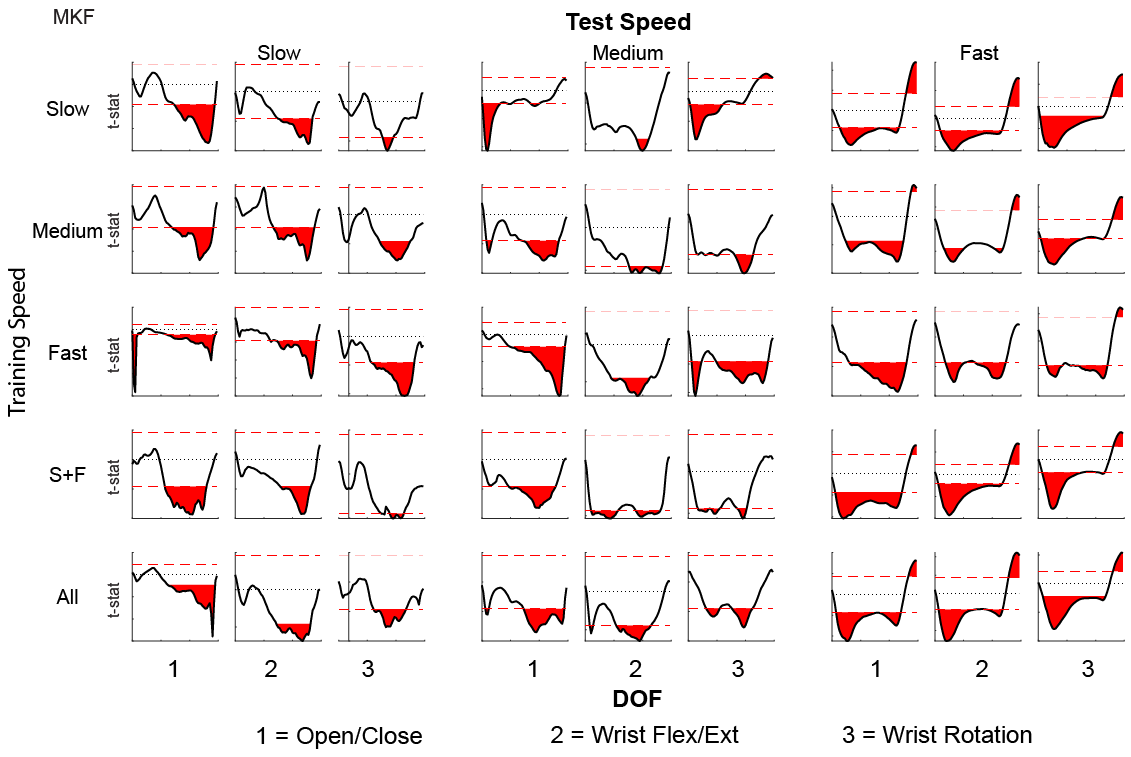


**Supplementary Fig. 2)** SnPM analysis of MKF-predicted kinematics revealed distinct temporal clusters of significant error during movement onset and offset across conditions. During slow test speeds, significant clusters were consistently observed during the return to rest phase across all training sets. Notably, training exclusively on fast speeds produced sharp onset overshoot error when tested on slow Open/Close movements. At medium test speeds, error localization varied by DOF, suggesting reduced task difficulty and better decoder alignment when using the MKF during medium test speeds. Fast test speeds elicited widespread, phase-spanning error regardless of training condition. Even under matched training and test speeds, MKF demonstrated onset and offset deviations, indicating poor generalization to dynamic transitions. Additionally, participants frequently failed to return fully to rest before the end of the movement, as indicated by positive t-statistic above the upper trajectory threshold near movement offset. T-statistics (black) compare predicted to true trajectories, with significant clusters (α = 0.05, corrected) shown in red.


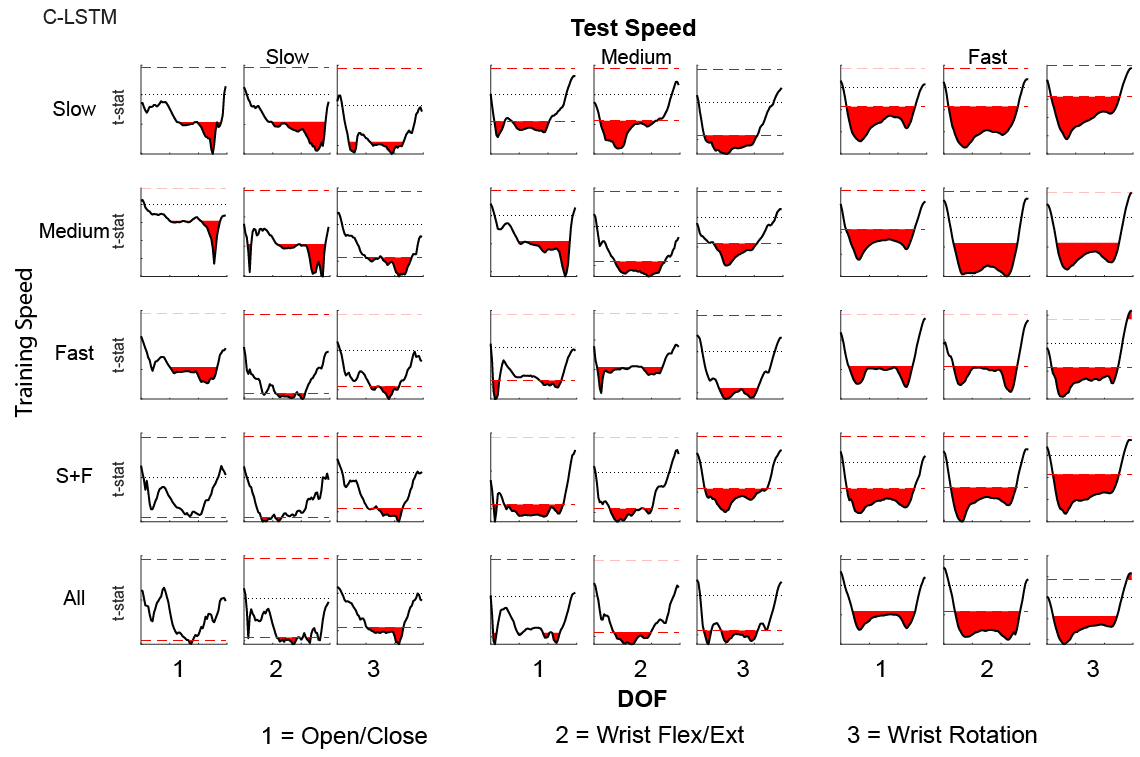


**Supplementary Fig. 3)** SnPM results for C-LSTM-predicted kinematics across all training and testing conditions during the TTT reveal consistent clusters of significant error at movement onset and offset. During slow test speeds, prediction error was the smallest and most often localized to the offset. Medium test speeds exhibited variability in error timing across training speeds. Most notably, it seems that medium training did not outperform other algorithms on its own testing data. Fast test speeds resulted in widespread and persistent error throughout the movement. As other metrics have suggested, the LSTM trained on multiple testing speeds showed the least temporal-specific error compared to other training conditions across testing conditions. T-statistics (black) compare predicted to true trajectories, with significant clusters (α = 0.05, corrected) shown in red.

1. **Supplementary Methods**
2. Statistical non-Parametric Mapping

To quantify temporal decoding errors across trajectories, we conducted a one-dimensional statistical analysis using Statistical non-Parametric Mapping. Paired t-tests were computed between the predicted and true trajectories for each DOF, participant, and condition, and averaged per participant prior to group-level testing (N=6). Each time point yielded a t-statistic, producing a continuous 1D t-trajectory. We employed a non-parametric randomization procedure to define statistical significance, controlling for multiple comparisons using a cluster-level threshold derived from 10,000 permutations [1], [2]. Significant clusters were identified at α = 0.05 (family-wise error corrected), highlighting regions in time where prediction error consistently exceeded zero across participants.

1. Kalman Filter Training and Implementation

The first decoder was a KF. The formulation of the Kalman filter for control was originally described in Wu et al. [3], its adaptation for EMG control is described in Nieveen et al. [4], and the ad-hoc modification has been described in George et al. [5]. The Kalman Filter (KF) is an online recursive estimator that computes the optimal state estimates by propagating state predictions and updating them with new observations. This supplementary section outlines how the KF is trained, how its parameters are estimated using least squares and Minimum Mean Square Error **(**MMSE**)** formulations, and how it is applied in real-time for inference.

1. Training the Linear Gaussian Model

In the Kalman filter for EMG-based kinematic decoding, the state transition matrix $A$ models how kinematic states evolve over time, while the observation matrix $H$ maps states to expected EMG features. The process noise covariance $Q$ captures variability in state evolution, and the observation noise covariance $R$ represents uncertainty in the kinematics-to-EMG mapping. To train the Kalman filter, we estimate the system matrices from the training data. The objective is to maximize the joint probability of the latent states $X_{M}$ and observations $Z_{M}$:

$$p\left( X_{M},Z_{M} \right)=\left[ p\left( x_{1} \right)\prod_{k=2}^{M} p\left( x_{k} \mid x_{k-1} \right) \right]\left[ \prod_{k=1}^{M} p\left( z_{k} \mid x_{k} \right) \right]$$

Using linear Gaussian assumptions, the closed-form solutions are given by:

$$A=\left( \sum_{k=2}^{M} x_{k}x_{k-1}^{\top} \right)\left( \sum_{k=2}^{M} x_{k-1}x_{k-1}^{\top} \right)^{-1}$$

$$H=\left( \sum_{k=1}^{M} z_{k}x_{k}^{\top} \right)\left( \sum_{k=1}^{M} x_{k}x_{k}^{\top} \right)^{-1}$$

$$Q=\frac{1}{M-1}\left( \sum_{k=2}^{M} x_{k}x_{k}^{\top}-A\sum_{k=2}^{M} x_{k-1}x_{k-1}^{\top} \right)$$

$$R=\frac{1}{M}\left( \sum_{k=1}^{M} z_{k}z_{k}^{\top}-H\sum_{k=1}^{M} x_{k}z_{k}^{\top} \right)$$

The state transition matrix $A$ and the observation matrix $H$ are estimated using least squares regression:

$$A=X_{2}X_{1}^{T}\left( X_{1}X_{1}^{T} \right)^{-1}$$

$$H=ZX^{T}\left( XX^{T} \right)^{-1}$$

The noise covariances $Q$ and $R$ are obtained using a Minimum Mean Square Error (MMSE) formulation. They are defined as the covariance of the residuals from the state and observation equations:

$$Q=cov\left( x_{k}-Ax_{k-1} \right)$$

$$R=cov\left( z_{k}-Hx_{k} \right)$$

1. Real-Time Kalman Filter Updates

Once the model parameters $A$, $H$, $Q$, and $R$ are estimated from training data, the Kalman filter can be applied in real time using recursive update equations. Each iteration consists of two steps:

- - - - 1. *Time Update (Prediction Step)*

The system model propagates the previous state estimate $\hat{x}_{\left\{ k-1 \right\}}$ forward to time $t_{k}$:

$$\hat{x}_{k}^{-}=A \hat{x}_{k-1}$$

$$P_{k}^{-}=A P_{k-1}^{+} A_{k}^{T}+Q_{k}$$

where $\hat{x}_{k}^{-}$ and $P_{k}^{-}$ denote the a-priori state estimate and error covariance, respectively.

- - - - 1. *Measurement Update*

The observation $z_{k}$ is incorporated to correct the prediction. The updated state estimate and posterior covariance are:

$$\hat{x}_{k}=\hat{x}_{k}^{-}+K_{k}\left( z_{k}-H\hat{x}_{k}^{-} \right)$$

$$P_{k}=\left( I-K_{k}H \right)P_{k}^{-}$$

where $K_{k}$ is the Kalman gain, defined as:

$$K_{k}=P_{k}^{-}H^{\top}\left( HP_{k}^{-}H^{\top}+R \right)^{-1}$$

This update ensures the state estimate minimizes the mean-squared reconstruction error.

1. Ad-hoc Modifications

George et al. describes a nonlinear output transformation applied after the Kalman filter update to make the decoder more intuitive for prosthetic use [5]. The core idea was to enforce a dead-zone threshold around rest and then rescale the output above threshold so that the dynamic range was preserved. The modified output equation they used is:

When $Output > Threshold$

$$Modified Output = \frac{\left( Output\cdot Gain \right)-Threshold}{1 - Threshold}$$

When $Output < Threshold$

$$Modified Output = 0$$

This modification deviates from the strict probabilistic KF but is critical in making control more stable, reducing unintended movements. In this study, a default threshold of 0.2 was used and the gain was unmodified (i.e., set to 1).

1. **Supplementary References**

[1] T. C. Pataky, “One-dimensional statistical parametric mapping in Python,” *Comput. Methods Biomech. Biomed. Engin.*, vol. 15, no. 3, pp. 295–301, Mar. 2012, doi: 10.1080/10255842.2010.527837.

[2] T. C. Pataky, M. A. Robinson, and J. Vanrenterghem, “Vector field statistical analysis of kinematic and force trajectories,” *J. Biomech.*, vol. 46, no. 14, pp. 2394–2401, Sep. 2013, doi: 10.1016/j.jbiomech.2013.07.031.

[3] W. Wu, Y. Gao, E. Bienenstock, J. P. Donoghue, and M. J. Black, “Bayesian Population Decoding of Motor Cortical Activity Using a Kalman Filter,” *Neural Comput.*, vol. 18, no. 1, pp. 80–118, Jan. 2006, doi: 10.1162/089976606774841585.

[4] J. Nieveen, M. Brinton, D. J. Warren, and V. J. Mathews, “A Nonlinear Latching Filter to Remove Jitter From Movement Estimates for Prostheses,” *IEEE Trans. Neural Syst. Rehabil. Eng.*, vol. 28, no. 12, pp. 2849–2858, Dec. 2020, doi: 10.1109/TNSRE.2020.3038706.

[5] J. A. George, T. S. Davis, M. R. Brinton, and G. A. Clark, “Intuitive neuromyoelectric control of a dexterous bionic arm using a modified Kalman filter,” *J. Neurosci. Methods*, vol. 330, p. 108462, Jan. 2020, doi: 10.1016/j.jneumeth.2019.108462.
